# Detection of Side Chain Rearrangements Mediating the Motions of Transmembrane Helices in Molecular Dynamics Simulations of G Protein-Coupled Receptors

**DOI:** 10.1016/j.csbj.2017.01.001

**Published:** 2017-01-14

**Authors:** Zied Gaieb, Dimitrios Morikis

**Affiliations:** Department of Bioengineering, University of California, Riverside 92521, USA

**Keywords:** Molecular dynamics, Change-point detection, Side chain reorganization, Helical domain motion, Intramolecular network, Membrane proteins, GPCR, GPCR computational modeling, GPCR allostery

## Abstract

Structure and dynamics are essential elements of protein function. Protein structure is constantly fluctuating and undergoing conformational changes, which are captured by molecular dynamics (MD) simulations. We introduce a computational framework that provides a compact representation of the dynamic conformational space of biomolecular simulations. This method presents a systematic approach designed to reduce the large MD simulation spatiotemporal datasets into a manageable set in order to guide our understanding of how protein mechanics emerge from side chain organization and dynamic reorganization. We focus on the detection of side chain interactions that undergo rearrangements mediating global domain motions and vice versa. Side chain rearrangements are extracted from side chain interactions that undergo well-defined abrupt and persistent changes in distance time series using Gaussian mixture models, whereas global domain motions are detected using dynamic cross-correlation. Both side chain rearrangements and global domain motions represent the dynamic components of the protein MD simulation, and are both mapped into a network where they are connected based on their degree of coupling. This method allows for the study of allosteric communication in proteins by mapping out the protein dynamics into an intramolecular network to reduce the large simulation data into a manageable set of communities composed of coupled side chain rearrangements and global domain motions. This computational framework is suitable for the study of tightly packed proteins, such as G protein-coupled receptors, and we present an application on a seven microseconds MD trajectory of CC chemokine receptor 7 (CCR7) bound to its ligand CCL21.

## 1 Introduction

Protein function is encoded into its dynamics as a large ensemble of conformations that can be grouped into distinct conformational states according to their function, free energy, and three-dimensional arrangement [1], [2]. These conformational states are accessed at different equilibrium sampling probabilities in response to outside perturbation such as ligand-binding, amino acid mutation, post translational modification, or environmental changes (pH, ionic strength, temperature, etc.) [3]. In many cases, ligand-free proteins that favor their inactive state, may still briefly sample their intermediate or active states [1]. However, external perturbations, such as ligand-binding, result in an equilibrium shift where the protein favors its active state.

As a mechanism to regulate its transitions and sampling of conformational states upon external perturbation, allosteric function plays an important role in transmitting information between distant functional sites of the protein [1], [2], [4]. To comprehend such mechanism, we must understand how the mechanics of protein structures emerge from the rearrangement of their constituent parts, specifically, side chain interactions within structured regions of proteins. Molecular dynamics (MD) simulation is one of the major techniques that has played a key role in studying protein dynamics at atomic level [2]. Several recent advances in enhanced sampling methods, simulation speed, and accuracy have allowed us to reach biologically relevant timescales that are sampled in the hundreds of nanosecond to microseconds and capture the transitioning of a protein between different states; and consequently, allow the study of allostery [2], [5], [6], [7]. Accordingly, several studies have explored the folding mechanism of a number of fast folding proteins [8] and captured protein state transitions [9], [10]. To extract biologically-relevant protein motions, long MD simulations have been analyzed through manual and visual inspection of large biological datasets of inter-atomic distance and Cartesian coordinate time series [7], [9], [10], [11], [12], [13], [14]. These extracted protein motions have consisted of abrupt changes in intramolecular interaction distance time series that show a transition between two stable inter-residue distances and the collective motion of many residues in different domains of the protein (transmembrane helices in our case). Despite the major advances in our understanding of protein dynamics, the MD analysis scientific community has not yet reached a consensus method to extract biologically-relevant conformational changes in proteins.

Many MD analysis tools have been developed, but still come short in detecting all relevant side chain and backbone rearrangements. Widely used methods involve the detection of global conformational changes, and include principal component analysis (PCA) and dynamic cross-correlation (DCC) applied to the three-dimensional Cartesian coordinates of simulated protein structures [15], [16], [17]. PCA, which is used to extract the dominant collective protein motions, tend to neglect less-dominant collective motions that are critical to unravel the complex details orchestrating protein transitions between conformational states. A heat map generated through DCC of aligned atomic Cartesian coordinates results in critical protein motions with low correlation coefficients (less than 0.6) due to noise introduced by atomic fluctuations and superimposition of the atomic coordinates, making it difficult to distinguish between false positives and false negatives [9]. Other methods revolve around the detection of abrupt changes in spatiotemporal data comprising of inter-atomic distances or three-dimensional coordinate time series [18], [19], [20]. The most recent method, SIMPLE, is designed to favor the detection of collective change-points, depending on a sensitivity parameter [20]. Despite the advances in event detection made possible by SIMPLE, this method still comes short in detecting all relevant side chain and backbone rearrangements. Depending on the sensitivity parameter used, many critical protein motions can either be obscured by the large number of detected change-points (large number of false positives) when using a low sensitivity parameter, or omitted (large number of false negatives) when using a high sensitivity parameter.

Due to the aforementioned challenges in biological event detection, many studies rely on manual and visual analysis of MD data [20]. These measures are non-systematic, are labor intensive, and may not provide a complete analysis due to the overwhelming amount of data output by the MD simulations. Systematic detection of protein motions is a critical step in understanding the molecular mechanism of protein allostery and is a challenging problem for many reasons. First, MD simulations output an insurmountable amount of dynamics information that can be daunting to analyze due to the high fluctuating and complex nature of protein dynamics. Second, side chain and domain rearrangements have very different dynamics behaviors, where amino acid residue side chains involve more fluctuations and sporadic movements than the larger domain movements of the protein [21]. Third, functional side chain rearrangements are subtle and manifest themselves as a single inter-residue interaction rearrangement that can be obscured by the several fluctuating and unstable inter-residue interactions. These challenges have prompted a need to reduce the large simulation data into a compact representation of the dynamic conformational space of biomolecules to guide scientists in their analysis of the complex MD simulation data.

In this work, we reduce the protein dynamics to its constitutive dynamic components. To carry their dynamics, proteins involve two major types of motions: side chain and global domain conformational changes. These motions constitute the dynamic components that facilitate the transmission of signals between distant sites in a protein [1], [2]. In the framework presented here, we start by screening for side chain rearrangements and global domain motions separately using Gaussian mixture models (GMMs) and DCC, respectively. All extracted components are then projected into a network based on their inter-component absolute average DCC coefficient and compartmentalized into different communities of correlated dynamics. The different network communities decompose the protein dynamics into its constitutive dynamic behaviors that are localized to different sectors of the protein, and comprise of side chain distance time series that are correlated (or anti-correlated) to the global domain motions of the protein. To illustrate the application of our computational framework, we apply our method to a previously published MD trajectory of a chemokine ligand, CCL21, bound to CC chemokine receptor 7 (CCR7) (Gaieb et al. REF). Essentially, our method reduces the dynamic interaction space of G protein coupled receptors (GPCRs) to a manageable space composed of protein sectors with different dynamic behaviors. The communities of dynamic components present a unified picture of the complex behavior of the protein and will guide the user to further analyze the subgraphs and communities to provide an understanding of how side chain rearrangements mediate the global motions of the protein, which eventually facilitates transitioning between functional states.

## 2 Materials and Methods

**Fig. 1:**
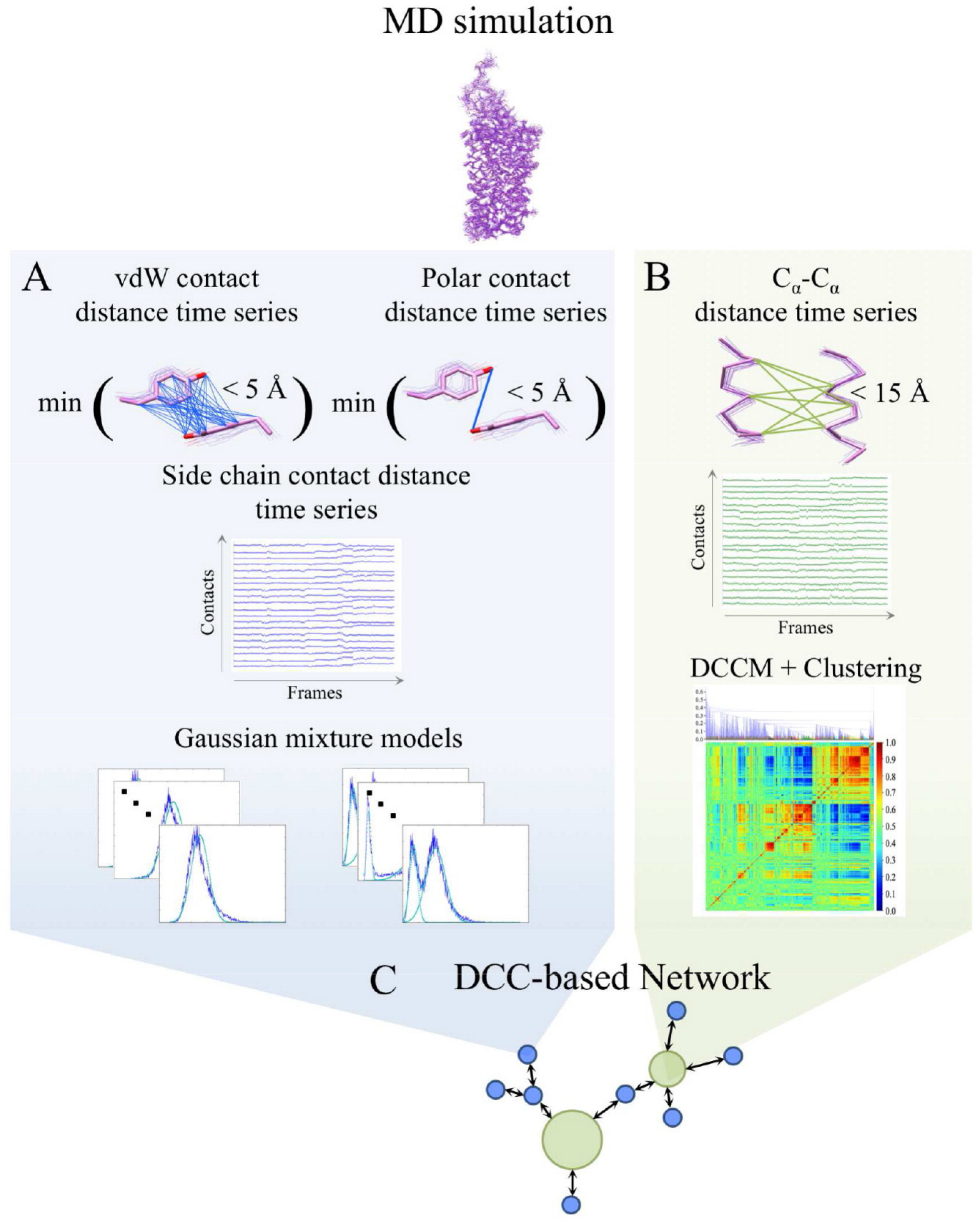
Schematic of our computational framework to extract coupled side chain rearrangements and global domain motions in proteins. (A) Van der Waals and polar interactions that sample a maximum distance of 5 Å during the simulation are used to calculate distance time series from the MD simulation 3-dimentional coordinate data. The minimum distance between all side chain or polar atoms are used to extract inter-residue side chain distance time series. Probability density of each time series are fitted to a GMM to extract side chain interactions that undergo rearrangements during the simulation. (B) C_**α**_-C_**α**_ interactions that sample a maximum distance of 15 Å during the simulation are used to calculate the C_**α**_-C_**α**_ distance time series. A DCC matrix of all pairwise C_**α**_-C_**α**_ distance time series are clustered and clusters with a minimum coefficient of 0.95 are extracted as domain motions of the protein. (C) Side chain rearrangements (blue nodes) and domain motions (green nodes) of the protein are considered dynamic components of the protein and are input into a DCC-based network to relate the two components to each other. Network connections are based on the correlation coefficients of pairwise dynamic components which are calculated as the average DCC coefficient of the pairwise time series belonging to each component.

Our computational framework is designed to systematically reduce the MD Cartesian coordinate time series of GPCRs to a few communities composed of coupled dynamic components (Fig. 1). This is done by first extracting side chain rearrangements and global domain motions from the protein's MD simulation trajectory.

Side chain rearrangements are often localized to a single inter-residue side chain interaction, which could be obscured by global domain motions when extracted from a large MD data set of inter-atomic distance time series. Therefore, both dynamic components, side chain (Fig. 1A) and backbone dynamics (Fig. 1B), are extracted separately using different methods: GMMs and DCC, respectively. Given the dynamic nature of proteins, only a fraction of the protein's extracted side chain dynamics is considered to contribute to regulating the global protein dynamics. Therefore, side chain rearrangements (Fig. 1A) are further reduced by extracting those that are correlated to the global domain motions (Fig. 1B). This is done by projecting all dynamic components into a network that is connected based on the absolute average inter-component correlation coefficient and then categorized into different communities, where domain motions and side chain dynamics within the same community show correlated time series (Fig. 1C).

### 2.1 Detection of Side Chain Contact Rearrangements From MD Simulations

**Fig. 2:**
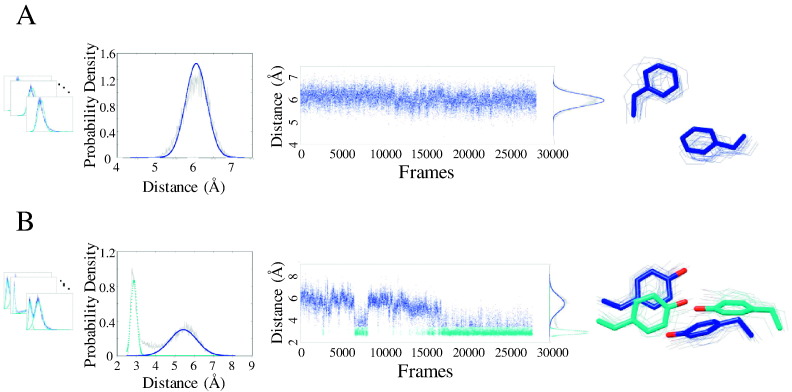
Examples of side chain distance probability densities fitted using GMM. (A) Side chain distance probability densities fitted by unimodal distributions show a stable inter-residue interaction through the majority of the simulation. (B) Side chain distance probability densities fitted by multimodal distributions represent inter-residue interactions that undergo rearrangements during the simulation. The cyan and blue colors represent the Gaussian distribution sampled around 2.7 Å and 5.5 Å, respectively.

Extracting all side chain rearrangements from MD simulations involves the identification of side chain interactions that experience abrupt and persistent changes in their distance time series, indicating a transition between substates. We extract such inter-residue interactions by fitting a GMM to the probability density of each interaction distance time series. GMMs are weighted sums of Gaussian densities and are used here as a parametric model of the probability density function of inter-residue time series (Gaussian densities are implemented in *scikit*-*learn*, a machine learning package in python) [22]. Stable non-varying interactions show a unimodal distribution (Fig. 2A), and multi-substate interactions show multi-modal distributions (Fig. 2B). The optimal number of Gaussians was efficiently determined using the Bayesian information criterion using *scikit*-*learn* [22], and GMM parameters were estimated using the iterative expectation-maximization algorithm, where the number of Gaussians is predetermined. This section of the computational framework is designed to systematically extract all interactions that show contact formation and breaking at any point during the simulations, as such contacts can be deemed critical in mediating global domain motions. GMMs are fitted to all distance time series representing van der Waals and polar interaction (listed below) distances between interacting side chain residues. Interacting residues used to calculate the distance time series are at least three residues apart in sequence and came into contact (a distance of at least 5 Å between all non-hydrogen side chain atoms) at any point during the simulation. To ensure complete formation and breaking of the side chain contacts, we calculate the inter-residue side chain distance time series using the minimum distance between all non-hydrogen side chain atoms of each of the amino acids. Similarly, polar interactions are also calculated using the minimum distance between all non-hydrogen polar head group atoms of interacting polar amino acids (atoms N_**ε**_, C_**ζ**_, N_**η1**_, or N_**η2**_ for R; atoms C_**γ**_, O_**δ1**_, or N_**δ2**_ for N; atoms C_**γ**_, O_**δ1**_, or O_**δ2**_ for D; atom S_**γ**_ for C; atoms C_**δ**_, O_**ε1**_, or N_**ε2**_ for Q; atoms C_**δ**_, O_**ε1**_, or O_**ε2**_ for E; atoms C_γ_, N_**δ1**_, C_**ε1**_, N_**ε2**_, or C_**δ2**_ for H; atom N_**ζ**_ for K; atom O_**γ**_ for S; atom O_**γ1**_ for T; atom N_**ε1**_ for W; atom O_**η**_ for Y). All distance time series probability density functions are fit with a GMM to identify the number of substates that each interaction is sampling.

Distance time series with unimodal GMMs are considered to be stable during the simulations, contributing to the structural stability (robustness) of the protein. On the other hand, multi-modal GMMs are amongst the dynamic components of the protein and contribute to the protein's conformational transitions between different functional states.

### 2.2 Detection of Global Domain Motions Through DCCM

Global domain motions in proteins involve the collective motion of backbone atoms and aid in the transitioning of the protein between different functional states. This part of the computational framework entails the detection of these motions as a collection of highly correlated inter-C_**α**_ distance time series.

**Fig. 3:**
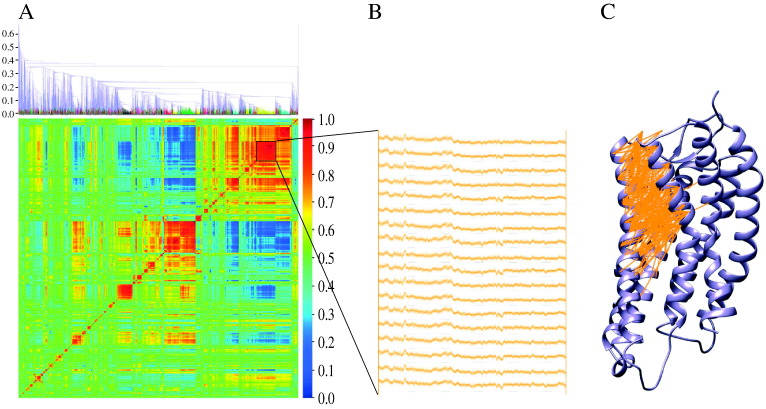
DCC heat map of pairwise C_**α**_-C_**α**_ distance time series are clustered using hierarchical clustering. (A) The clustering dendrogram is reported above the DCC heat map. The DCC coefficient is used as the distance calculated between two clusters and shown as the y-axis of the dendrogram. Each color of the dendrogram represents a different cluster of time series that are correlated at a cutoff DCC coefficient of 0.95. Due to the large number of C_**α**_-C_**α**_ distance time series, only time series within the extracted clusters are shown in the DCC heat map. (B) An illustration of the time series within the highlighted cluster in (A). (C) An example of molecular graphics demonstrating the interacting residues involved in the domain motions between TM5 and TM6 illustrated in the highlighted cluster in (A). Each connection involves two C_**α**_ whose distance time series is within the highlighted cluster in (A).

All alpha carbon interactions (at least three residues apart in sequence) within 15 Å at any point of the simulation are extracted, and all distance time series representing theses interactions are calculated. Pairwise dynamic cross-correlation of all distance time series are clustered based on their correlation coefficient and clusters with at least 0.95 correlation coefficient are extracted (Fig. 3A, B). Each cluster is a set of highly correlated time series that are localized to distinct protein sectors that exhibit different dynamic behaviors (Fig. 3C). The algorithm for hierarchical clustering used is provided in the *SciPy* library (scipy.cluster.hierarchy.linkage), and is performed on a condensed distance matrix using the Nearest Point Algorithm [23]. The condensed distance matrix is defined as a pairwise correlation coefficients matrix between extracted time series and is returned by the scipy.spatial.distance.pdist function.

The use of distance time series (rather than Cartesian coordinates) presents various advantages in molecular dynamics simulation analysis. Apart from reducing the dimensionality of the time series used (from three-dimensional Cartesian coordinates to one-dimensional distance time series), the translation and rotation of the whole protein during the MD simulations can be ignored and, therefore, structure superimposition (alignment) can be omitted. These improvements allow us to accentuate the changes in the global structure of the protein and attenuate the effects of atomic fluctuations seen when using the Cartesian coordinates. Thus, clusters with high DCC coefficient better portray the global domain dynamic behavior of the protein.

### 2.3 Network of the Protein's Dynamic Components

**Fig. 4:**
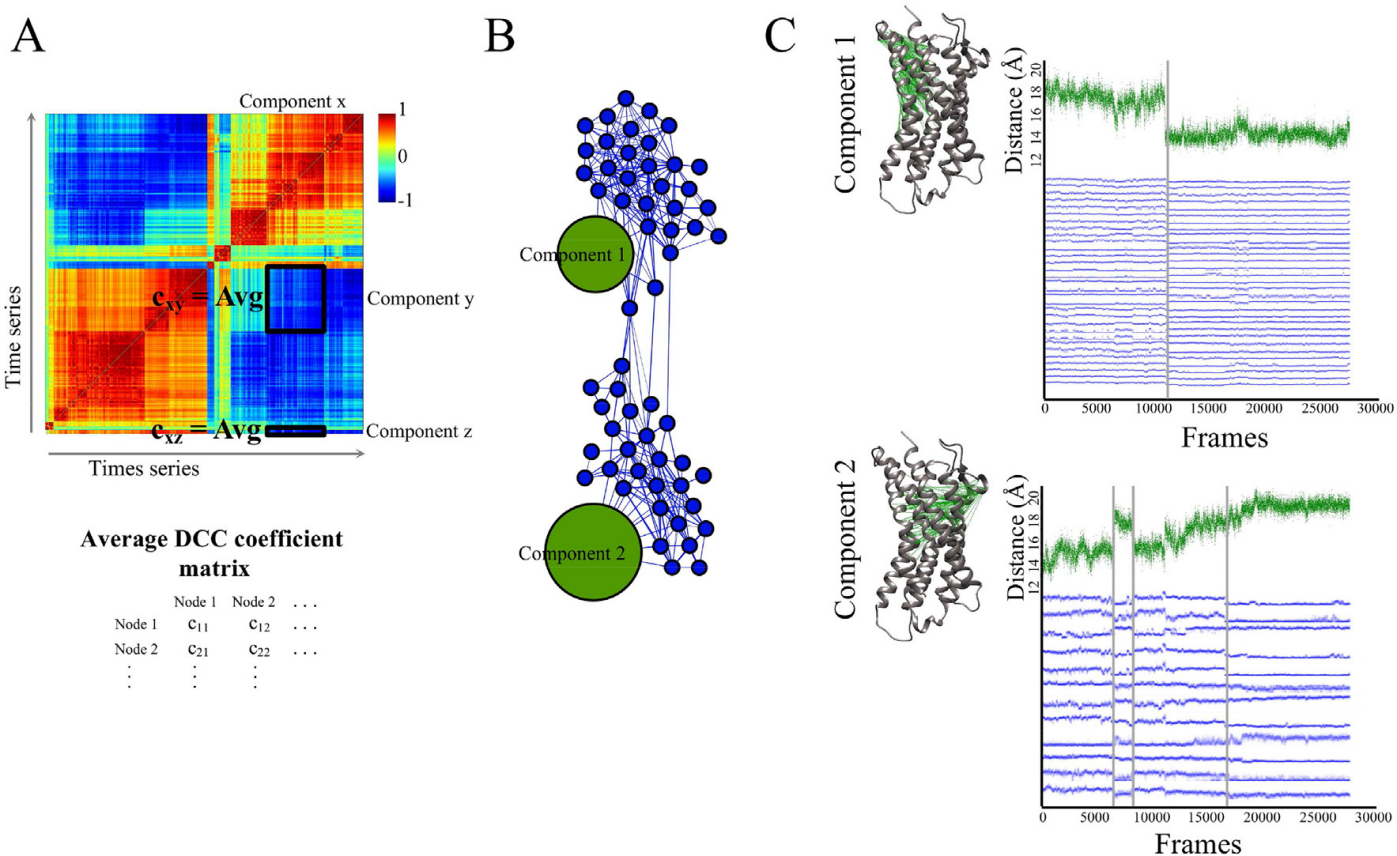
DCC-based network illustration of the protein's dynamic components. (A) Correlation coefficients of pairwise dynamic components are calculated as the absolute average DCC coefficient of the pairwise time series belonging to each component as illustrated on a sample DCC heat map. Average correlations are calculated between pairwise domain motions (components x and y), between pairwise side chain rearrangement time series (component z) and across both components (components x and z). Average DCC coefficient matrix is generated for all pairwise dynamic components. (B) The network is built from a subset of the time series extracted from the MD simulation of CCL21-bound CCR7. The network is composed of two communities that are centered around domain motions labeled as component 1 and component 2. Network nodes represent the dynamic components extracted from the subset time series data and are colored blue for side chain rearrangements and green for domain motions. The size of each node is proportional to the number of time series the node represents. Edges connecting the dynamic components are based on the absolute average pairwise DCC coefficient of the time series involved in each of the components. Edges are drawn between dynamic components of a minimum coefficient of 0.75. C_**α**_-C_**α**_ distance. (C) Time series that comprise each of components 1 and 2 are projected into the molecular graphics of CCR7 and labeled accordingly. Components 1 and 2 represent domain motions in a protein and are constituted of several highly correlated C_**α**_-C_**α**_ distance time series. A sample time series from each of the domain motion components is shown in green. Blue time series are side chain time series for each of the blue nodes within each of the communities centered around components 1 and 2. All time series show coupled abrupt changes within each of the domain movements highlighted in grey. The network was built using Gephi [27].

To assess coupling between side chain rearrangements and global domain motions, these dynamic components of the protein are projected into a static network and classified into communities, using *igraph* [24]. We create a DCC-based network connecting the dynamic components of the protein (Fig. 4), extracted in the previous sections and illustrated in Fig. 2, Fig. 3. In the network (Fig. 4B), the blue and green nodes represent side chain and backbone interactions, respectively; and edges connect correlated components with a minimum absolute average correlation defined by the user (The network in our CCR7 case was constructed using a minimum average absolute correlation of 0.75).

Average correlation coefficients of pairwise dynamic components are calculated as the absolute value of the average DCC coefficient of the pairwise time series belonging to each component (Fig. 4A). The absolute average DCC coefficient matrix is generated for all the pairwise dynamic components and is then projected into the network where components are connected based on an average DCC coefficient cutoff (Fig. 4B). The absolute average correlation coefficient cutoff represents the degree of coupling between dynamic components, and can be adjusted to account for weak couplings between the fast side chain rearrangements (picosecond and nanosecond timescales) and the slow global motions of protein domains (microsecond and nanosecond timescales) (Fig. 4C, upper panel). In addition, while rearrangements in side chain interaction are manifested as abrupt changes in the distance time series, the global domain motion experiences more incremental changes that span hundreds of nanoseconds and can still be accounted for by also adjusting the cutoff absolute average DCC coefficient (Fig. 4C, lower panel). The size of each node is proportional to the number of distance time series each dynamic component (node) represents, where global dynamic components involve many time series, while side chain rearrangements are characterized by one distance time series (Fig. 4). Network communities are detected based on edge betweenness using the community_edge_betweenness method in *igraph* [24]. Each community is composed of side chain and backbone dynamics that are coupled to each other and represents the dynamic behavior of a protein subdomain that is encoded in its side chain rearrangements and global domain motions (Fig. 4C).

### 2.4 Network Community Visualization Using Molecular Graphics Visualization Tools

MD simulations provide an insurmountable amount of dynamic information due to the high fluctuating and complex nature of protein dynamics. Here, the extracted communities reveal to be useful in reducing the MD data to its functional dynamic behavior, where each community is composed of coupled side chain rearrangements and global domain motions. These communities can be output into a protein data bank (PDB) file format to visualize the residues that make up the dynamic components of the community and each time series belonging to the dynamic components can be output as a pseudobond connecting two representative atoms of the time series' corresponding residues (Fig. 3C). This allows for better visualization and further analysis of the residues involved in mediating the allosteric communication within the protein.

## 3 Results and Discussion

### 3.1 Application to Molecular Dynamics Simulation Data

We apply our computational framework to previously published 7 μs-MD trajectory where we analyzed the simulations to understand the mechanism by which information is transmitted in CCR7 when bound to its agonist ligand, CCL21 [11]. We have determined key conformational changes that act as molecular switches and facilitate the transitioning of the receptor between its different states by inducing global motions of its transmembrane domain (TMD) helices [11]. The simulation dataset of CCR7 was originally analyzed through manual and visual inspection of a large set of distance time series and generic summary quantification, such as root mean square deviation (RMSD), principal component analysis (PCA), and comparison of the inter-residue mean distances between different time segments. Such non-systematic measures are very labor intensive and may not provide a complete analysis due to the overwhelming amount of the data output by the MD simulations. Nonetheless, we were able to detect a series of molecular switches that are mediated by various ligand-induced allosteric events. These molecular switches involve three tyrosine residues (Y112^3.32^, Y255^6.51^, and Y288^7.39^), three phenylalanine residues (F116^3.36^, F208^5.47^, and F248^6.44^), and a polar interaction between Q252^6.48^ and R294^7.45^ in the TMD of CCR7 [11]. Molecular events within these switches are coupled with global movements in the receptor's TM helices and contribute to the transitioning of the receptor to distinct states.

In our test case here, we apply our computational framework to the CCL21-bound CCR7 MD simulation data [11]. Using a distance cutoff of 5 Å, a total of ~ 1200 inter-residue side chain distance time series were imported and fit to a GMM in order to systematically extract all multi-modal distance probability densities. The selected contacts reduced our data set to ~ 600 time series. However, the majority of these contacts comprises of independent side chain rearrangements that do not contribute to the protein's major motions, and only a fraction of these multi-modal contacts will remain in the final network of coupled dynamic components. The second part of our computational framework focused on extracting the receptor's global domain motions using inter-residue C_**α**_ distance time series with a cutoff of 15 Å. A pairwise DCC matrix was generated for ~ 6000 distance time series, and then clustered at a DCC coefficient cutoff of 0.95. The high DCC cutoff generated clusters with highly correlated distance time series that involve structurally adjacent amino acids. This part of the computational framework generated ~ 1000 clusters which included multiple clusters of more than one hundred time series (clusters containing a large number of time series represent a large number of residues involved in a global domain motion). After calculating all pairwise absolute average DCC coefficients between all dynamic components and projecting our data onto a DCC-based network, all dynamic components were then reduced to six communities with different dynamic behaviors that make up the orchestrated complex motions involved in transitioning CCR7 between two different states [11].

**Fig. 5:**
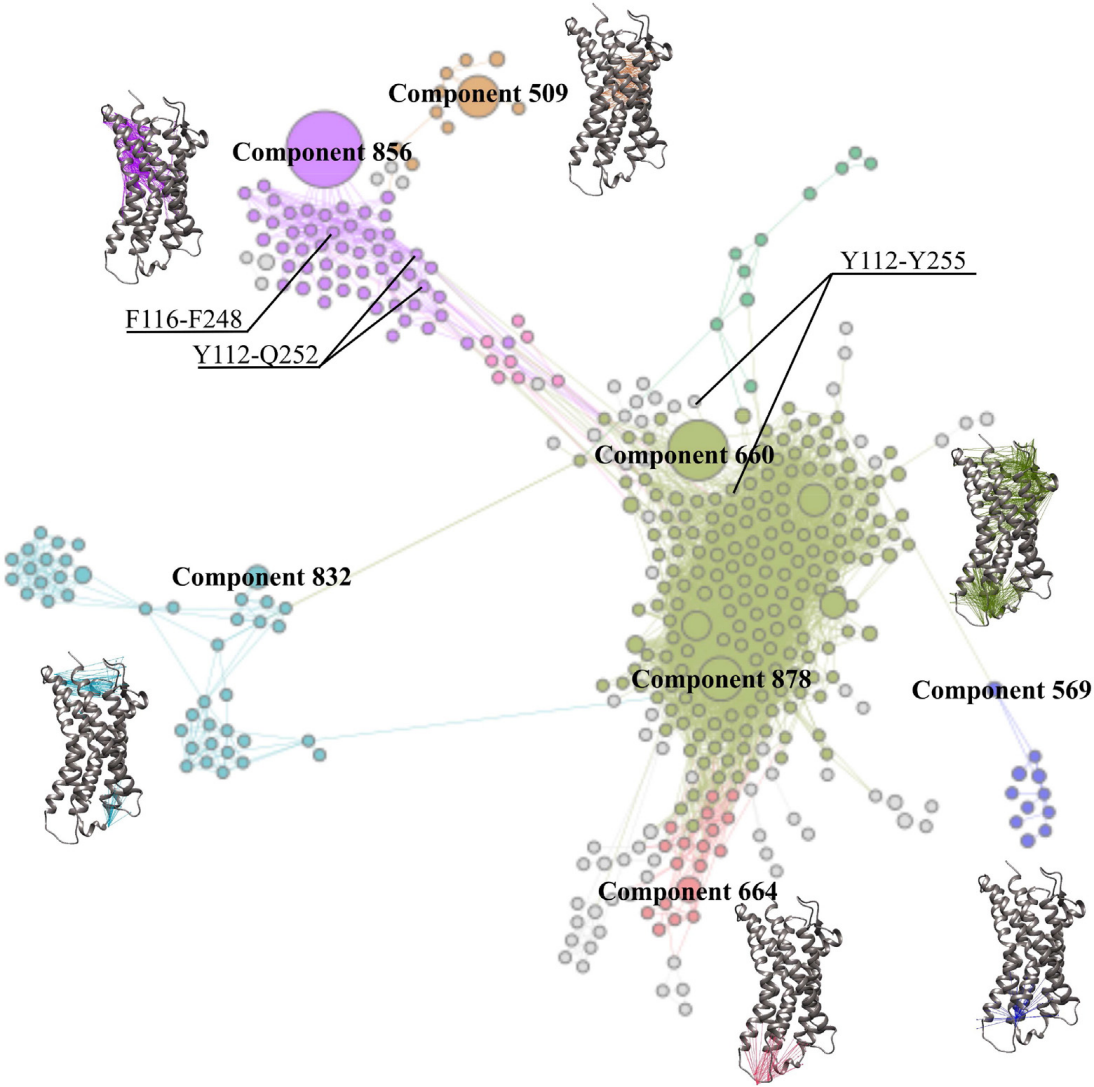
A DCC-based network of the full CCL21-bound CCR7 MD simulation dataset. Network communities are colored differently and dynamic components representing domain motions are projected into a molecular graphics in which connections are colored according to the community they belong to. Previously determined molecular switches (F116-Q252, Y112-Q252, Y112-Y255) are labeled accordingly in the network [19].

Using our computational framework, we systematically decomposed the protein dynamics into different sectors (subdomains) that show varying dynamic behaviors (Fig. 5). Our method reduces the protein dynamic interaction space of ~ 8000 time series into a network of 280 nodes representing side chain dynamic components and 127 nodes representing global domain dynamic components (node sizes is proportional to the number of time series representing the dynamic components). Each community is composed of a few nodes that represent the main global motions of CCR7 and several nodes that represent side chain rearrangements. The network is decomposed into six communities that present a unified picture of the complex behavior of the protein's helices and loops. Each community contains all coupled dynamic components of the simulation and can be further analyzed to extract critical molecular switches that coordinate protein dynamics. Molecular switches consist of side chain rearrangements that switch controllably between two or more stable states in response to perturbations, and can be challenging to isolate from MD simulation data due to the complex dynamics of protein. Thus, summarizing and categorizing all dynamics data into a network will provide a clear picture of the large MD data sets of GPCRs that can be further analyzed using the extracted small set of communities. Analysis can be performed through manual and visual comparison between conformational states of each community, as performed by Vanatta et al. [25]. Each protein sector can also be clustered into states to extract control variables that could select for one or more of the conformational states using the Jensen-Shannon divergence statistics, as described by Fenley et al. [26]. Within our test case network, previously determined molecular switches highlighted in Fig. 5 (F116-F248, Y112-Y255, and Y112-Q252) were detected through visual and manual comparison of CCR7 conformational states [11]. These molecular switches belong to different communities centered around global motions of the receptor helices, which demonstrates their coupling to different global dynamic components of the receptor.

## 4 Concluding Remarks

This computational framework focuses on reducing the MD simulation data into a more manageable dynamic interaction space by mapping the GPCR dynamics into an intramolecular network of dynamic components composed of coupled side chain rearrangements and global conformational changes. This is done through the detection of side chain contacts with multi-modal probability density function and global domain motions manifested as clusters of highly correlated inter-residue C_**α**_ distance time series. Community detection in a DCC-based network of all extracted components correlate the side chain contacts to the domain motions in order to map all the different dynamic components of the protein into various communities of different dynamic behaviors.

As a proof of concept, this method was applied to a MD simulation of CCR7 to systematically detect the different protein sectors responsible for mediating the complex motions of its helices. Ultimately, our computational framework reduces the overall behavior of the protein to a set of communities composed of coupled side chain and global dynamic components. This method provides a reduced and more manageable dataset, where each community representing a separate protein sector can be further analyzed separately.
